# Supplementary material for: Junction Mapper is a novel computer vision tool to decipher cell–cell contact phenotypes
Source: eLife. 2019 Dec 3;8:e45413. doi: 10.7554/eLife.45413 (PMC7034980; doi:10.7554/eLife.45413)
Supplement: Supplementary file 4. [file elife-45413-supp4.docx]

**Minimizing user bias**

User bias can influence the quantification data and has a stronger influence on the measurement outcomes of images with disrupted cell-cell contacts. Junction Mapper users (especially those working on the same project) should set up and follow closely clear criteria for setting parameters such as corner positioning, dilation and thresholding. The criteria should be revised when users encounter a challenging or ambiguous image, e.g. when deciding how to set corners or create the skeleton between perturbed junctions. Since the secondary parameters are more robust measurements they should be evaluated preferentially when analysing challenging data sets.

We recommend the following guidelines based on our experience with analysing the junctions in keratinocytes, HUVEC cells, and cardiomyocytes:

| **Step in the process** | **Heuristics for minimizing user bias and/ or recommended user guideline** |
| --- | --- |
| **Edge Detection** | **Staining**: The staining protocol should be optimized to minimize cytoplasmic and non-specific staining.  **Image quality**: Highest possible quality of image (ideally taken using a confocal microscope) should be used.  **Peak signal-to-noise ratio (PSNR)**: A suitable signal-to-noise ratio is necessary to ensure enough contrast to differentiate staining at junctions from the cytoplasm for automated cell edge detection. We recommend PSNR of at least 22dB (Figure2-figure supplement 3). |
| **Produce and finesse the binary edge map** | Any discrepancies between algorithm-detected cell outline and true path of the junctions on the image must be corrected manually. The incorrect portion of the junction needs to be deleted using the “Remove” tool and a new line drawn through the centre of the junction (or the suspected centre of the junction based on adjacent fragments for disrupted junctions) with the “Add” tool. The line location should then be fine-tuned to go through the brightest pixels by applying “Local Maxima for Edges” with a small window size, e.g. 3 pixels. The resulting fragmented lines are then reconnected by applying “Dilate” enough times to connect all fragments and then “Skeletonise”.  **Zig-zag junctions**: In images of cells with zig-zag junctions (such as those in thrombin-treated endothelial cells, Figure 7A), the edge map identified by Junction Mapper is somewhat linear and thus it is not an exact representation of junction marker staining. This caveat does not prevent the analyses, but rather limits the subset of parameters to those that derive their quantification from area- rather than length-based measurements. |
| **Select cells to be analysed** | **Focal planes**: Blurry parts of images (different focal planes or out of focus areas) should not be analysed as this would result in inaccurate measurements. This can be prevented (and number of suitable cells increased) by taking Z-stacks instead of single-focal plane images. For Junction Mapper to quantify from Z-stack images, individual sections would need to be extracted and analysed separately. Alternatively, a projection of all sections can be analysed.  **Colony edge**: At a junction at the very edge of the colony, there is a missing corner as it is a border between two cells, rather than three. The automatic corner detection might not work correctly and the corner needs to be set manually. The cell outline at colony edges might need more manual correction. For some research questions, these junctions may be excluded as part of the quality control.  **Expression of exogenous proteins**: When analysing cells induced to overexpress a protein of interest, cells of similar expression level, as judged by staining intensity, should be analysed. Cells expressing too little or too much exogenous protein should not be analysed, particular when cell retraction is observed. |
| **Define corners between cells** | **Corners**: Any discrepancies between the automated corner detection and perceived corner location on visual image inspection should be corrected manually. Corners should be identified as the meeting point between junctions of three or more cells (Figure 3) or the end of the joined interface between two cells at a colony border. If corners are not covered by junctional staining, they should be identified by estimating the likely meeting point of junctions from neighbouring cells. Addition of a cytoplasm marker (dye or F-actin staining for example) helps defining where the contacting interfaces are likely to meet. Disparate corner position when contacts are disrupted is one of the key influences of user-bias. |
| **Thresholding** | **Thresholding**: Thresholding affects both length- and area-based parameters as it directly determines the number of pixels included in the analysis (Figure 3-figure supplement 3). The best threshold should be determined on a set of control images that covers the range of staining intensities in the control data set. Optimal threshold values detect all junctional staining without including background signal in slightly brighter image areas and without losing all junctional staining in dimmer staining areas. Depending on the aim of the analysis, different users analysing the same data set, might want to consider agreeing on threshold values to minimise user bias. |
| **Edge Map dilation** | **Dilation**: Dilation affects the area-based parameters, as it determines the number of pixels considered to belong to a junction. The dilation value is chosen to include all (or nearly all) of the junctional staining while avoiding significant contribution of cytoplasmic staining (especially with larger dilation values; Figure 3-figure supplement 2). This can be challenging for cells with very uneven junctions, such as cardiomyocytes. Different users should agree on a dilation value when analysing the same data set. |
| **Measure different parameters at cell-cell contacts** | **Length-based parameters** (Interface Contour, Fragmented Junction Contour, Junction Contour): These parameters do not consider the width of individual junctions and are thus not suitable to describe the entire phenotype where junction staining is thick. Length-based parameters (both primary and secondary parameters) are not suitable for analysis of cells with jagged junctions, as the edge map obtained is relatively linear and does not recognize the zig-zag pattern, thereby underestimating the contour.  **Primary parameters** are useful to show extension or retraction of the contacting interface/junctions, increased fragmentation of the staining of junction marker or fluctuations of global staining intensity at cell-cell contacts.  **Secondary parameters** Interface Linearity Index, Coverage Index, Interface Occupancy, Intensity per Interface Area and Cluster Density parameters are less prone to user bias (Supplementary file 3) as they are normalized to the corresponding junction or interface length/area |
| **Quality control** | When all selected cells of an image have been measured, the data have to be cleaned up, i.e. remove junctions that are out of focus, in areas with problematic staining and artefacts or if strong cell retraction is present, with no contacting interface. In addition, the same junction may have been quantified twice from neighbouring cells and hence such duplicated data must be removed from analyses. The same criteria for junction exclusion should be agreed and made consistent in the analyses by different users. |
