# Supplementary material for: Junction Mapper is a novel computer vision tool to decipher cell–cell contact phenotypes
Source: eLife. 2019 Dec 3;8:e45413. doi: 10.7554/eLife.45413 (PMC7034980; doi:10.7554/eLife.45413)
Supplement: Transparent reporting form [file elife-45413-transrepform.pdf]

eLIFE
1st Floor
24 Hills Road
Cambridge CB2 1JP, UK
P 01223 855340
W elifesciences.org
T @elife

eLife's transparent reporting form

We encourage authors to provide detailed information *within their submission* to facilitate the interpretation and replication of experiments. Authors can upload supporting documentation to indicate the use of appropriate reporting guidelines for health-related research (see EQUATOR Network), life science research (see the BioSharing Information Resource), or the ARRIVE guidelines for reporting work involving animal research. Where applicable, authors should refer to any relevant reporting standards documents in this form.

If you have any questions, please consult our Journal Policies and/or contact us: editorial@elifesciences.org.

Sample-size estimation

- You should state whether an appropriate sample size was computed when the study was being designed
- You should state the statistical method of sample size computation and any required assumptions
- If no explicit power analysis was used, you should describe how you decided what sample (replicate) size (number) to use

Please outline where this information can be found within the submission (e.g., sections or figure legends), or explain why this information doesn't apply to your submission:

The total number of junctions measured in different experiments were (average amount per sample in each experiment is in brackets):

H-Ras expression – 771 junctions (257)
Src expression – 335 junctions (112)
Rac1 expression – 215 junctions (72)
CIP4 siRNA – 751 junctions (376)
EEF1A siRNA – 762 junctions (381)
VAV2 siRNA – 474 junctions (237)
Endothelial cell – 391 junctions (196)
Cardiomyocytes - 380 junctions (190)

The individual values quantified per sample are written inside the graphs in the different figures.

Replicates

- You should report how often each experiment was performed
- You should include a definition of biological versus technical replication
- The data obtained should be provided and sufficient information should be provided to indicate the number of independent biological and/or technical replicates
- If you encountered any outliers, you should describe how these were handled
- Criteria for exclusion/inclusion of data should be clearly stated
- High-throughput sequence data should be uploaded before submission, with a private link for reviewers provided (these are available from both GEO and ArrayExpress)

Please outline where this information can be found within the submission (e.g., sections or figure legends), or explain why this information doesn't apply to your submission:

Quality control is found in the Methods Section. Quality control of the data before quantification was as follows: junctions were excluded if they have blurry areas, artefacts or large gaps between cells. Regions were also excluded that contained (i) junctions of cells overlapping or on top of each other (different focal plane) (ii) multinucleated cells (iii) cells that were not fully surrounded by neighbours (i.e. at the border of the image or epithelial colony), (iv) junctions of cells overexpressing high levels of plasmids. As values are obtained per junction and a junction is shared by two cells, duplicated measurements of junctions are removed from the dataset.

For expression of different oncogenes, junctions were classified as those between (i) control cells (between two non-transfected cells), (ii) between two expressing cells (ee) or (iii) junctions shared by one expressing and a non-expressing cell (en). For the analyses of endothelial cells - junctions in blurry parts of the picture, artefacts or large gaps between junctions, junctions at the border of epithelial colony were excluded. For the cardiomyocytes, same criteria were applied for endothelial and the parts of the image where the staining seemed very scattered and chaotic, with no clear trace of a junction were also excluded.

A total of 4,080 junctions were quantified across different treatments (expression, siRNA or stimulus) and cell types using different batches of cells. Data compare different samples from one technical replicate for each treatment condition, with an average of 227 junctions for each sample performed in parallel.

The aim of the manuscript is to demonstrate the ability of the software to identify various parameters and identify disruption patterns characteristic of each type of stimulus. For some experiments, we validate previous quantification performed in the lab by alternative methods (i.e. siRNA experiments, intensity and % area parameters; Erasmus et al., 2016). For biological conclusions on specific phenotypes, more technical and biological replicates should be performed.

Statistical reporting

- Statistical analysis methods should be described and justified
- Raw data should be presented in figures whenever informative to do so (typically when N per group is less than 10)
- For each experiment, you should identify the statistical tests used, exact values of N, definitions of center, methods of multiple test correction, and dispersion and precision measures (e.g., mean, median, SD, SEM, confidence intervals; and, for the major substantive results, a measure of effect size (e.g., Pearson's r, Cohen's d)
- Report exact p-values wherever possible alongside the summary statistics and 95% confidence intervals. These should be reported for all key questions and not only when the p-value is less than 0.05.

Please outline where this information can be found within the submission (e.g., sections or figure legends), or explain why this information doesn't apply to your submission:

Normality test was performed in each dataset using Kolmogorov-Smirnov test, D'Agostino & Pearson omnibus normality test, and Shapiro-Wilk test. Data from Src^{Y527F}, H-Ras^{G12V} and Rac1^{Q61L} experiments were analysed using ANOVA with the Games-Howell post-hoc test from the 'userfriendlyscience' in RStudio. Despite the data being non-parametric, the larger sample size (>100) allows for the use of ANOVA with Games-Howell post-hoc test, which corrects for unequal sample sizes and variances between groups and for the data with non-parametric distribution. Pair-wise comparison was analysed with Wilcoxon matched paired test. Data with single treatment group and control group (this includes siRNA experiments in epithelial cells, cardiomyocytes and endothelial cells) were non-parametric and hence was analysed using the Mann-Whitney U-test in GraphPad PRISM.

Raw images are presented in each figure. Exact p-values are reported in each graph. Statistical methods are clearly described in the methods Section and in each relevant figure legend.

(For large datasets, or papers with a very large number of statistical tests, you may upload a single table file with tests, Ns, etc., with reference to sections in the manuscript.)

Group allocation

- Indicate how samples were allocated into experimental groups (in the case of clinical studies, please specify allocation to treatment method); if randomization was used, please also state if restricted randomization was applied
- Indicate if masking was used during group allocation, data collection and/or data analysis

Please outline where this information can be found within the submission (e.g., sections or figure legends), or explain why this information doesn't apply to your submission:

Groups were allocated according to experimental conditions to be compared. Each experiment was performed with its own controls to be compared to (i.e. non-targeting siRNA, unstimulated cells, or non-expressing cells). No masking was done during data collection or quantification. Cells were selected at random, but according to the criteria described above in quality control.

Additional data files ("source data")

- We encourage you to upload relevant additional data files, such as numerical data that are represented as a graph in a figure, or as a summary table
- Where provided, these should be in the most useful format, and they can be uploaded as "Source data" files linked to a main figure or table
- Include model definition files including the full list of parameters used
- Include code used for data analysis (e.g., R, MatLab)
- Avoid stating that data files are "available upon request"

Please indicate the figures or tables for which source data files have been provided:

The raw numerical data for each figure has been uploaded as source data (i.e. excel file). Table summaries are already provided in the main manuscript.
